# Case report and literature review of IgG4-related autoimmune pancreatitis secondary to pancreatic involvement of IgG4-related sclerosing disease

**DOI:** 10.3389/fmed.2026.1793667

**Published:** 2026-05-13

**Authors:** Rongchun Xing, Dan Yu, Yan Li, Hang Zhou, Chongyuan Chen, Mingzheng Hu

**Affiliations:** 1 The First College of Clinical Medical Science, China Three Gorges University, Yichang, Hubei, China; 2 Department of Hepatobiliary Surgery, Yichang Central People’s Hospital, Yichang, Hubei, China

**Keywords:** IgG4-related autoimmune pancreatitis, IgG4-related sclerosing disease, focal mass-forming type, pancreatic ductal adenocarcinoma, differential diagnosis, pancreaticoduodenectomy, case report

## Abstract

**Background:**

When IgG4-related sclerosing disease (IgG4-RD) involves the pancreas, it frequently manifests as focal mass formation. These lesions clinically and radiographically closely mimic pancreatic ductal adenocarcinoma, often leading to unnecessary invasive interventions. Clarifying the diagnostic difficulties in differentiating focal mass-forming IgG4-RD from malignancies is of great significance for optimizing initial clinical assessment and management workflows.

**Methods:**

This study reports the complete clinical course of a 70-year-old male patient who presented with a complaint of upper abdominal bloating, manifesting as a pancreatic head mass accompanied by a significant elevation of the tumor marker CA19-9 and biliary obstruction. Due to a high preoperative suspicion of malignancy and diagnostic challenges, the patient underwent a pancreaticoduodenectomy. Concurrently, this study conducted a systematic review and analysis of previous literature related to IgG4-RD presenting as focal pancreatic space-occupying lesions to explore the common diagnostic pathways and potential pitfalls in such cases.

**Results:**

The patient’s postoperative pathological and immunohistochemical examinations ultimately confirmed that the lesion was autoimmune pancreatitis secondary to IgG4-related sclerosing disease, with no malignant components identified. The literature review further indicated that, in the early stages of the disease when clear evidence of systemic involvement is lacking, the space-occupying effect on imaging often dominates the diagnostic pathway, obscuring its immune-mediated inflammatory nature.

**Conclusion:**

For patients with suspected pancreatic tumors, incorporating IgG4-RD into the routine differential diagnosis can serve as a beneficial relative reference clue. Provided that malignancy is strictly evaluated and excluded through methods such as endoscopic ultrasound-guided tissue biopsy, early recognition of the disease’s inflammatory characteristics combined with multidisciplinary assessment can facilitate the transition of treatment strategies from surgical resection to pharmacological management, thereby optimizing clinical decision-making and avoiding overtreatment.

## Introduction

IgG4-related sclerosing disease (IgG4-RD) is a systemic disease characterized by immune-mediated chronic inflammation and progressive fibrosis, capable of involving multiple organs and tissues (1). Its pathological features include dense lymphoplasmacytic infiltration, storiform fibrosis, and obliterative phlebitis, accompanied by a significant increase in the number of IgG4^+^ plasma cells (2). The disease exhibits significant heterogeneity in clinical presentation and imaging morphology depending on the organ involved, making early recognition of IgG4-RD difficult (3).

When IgG4-RD affects the pancreas, it can lead to IgG4-related autoimmune pancreatitis (IgG4-AIP). Unlike common inflammatory pancreatic diseases, IgG4-AIP does not always present with typical symptoms of acute or chronic pancreatitis (4). Clinically, patients frequently present with non-specific but concerning manifestations such as upper abdominal bloating, dull pain, and obstructive jaundice, which are key symptoms that initially drive the diagnostic approach. Instead, it more frequently comes to clinical attention due to morphological changes in the pancreas and secondary biliary compression (5). While some patients present with diffuse pancreatic enlargement, in a significant proportion of cases, the lesion is localized to a specific part of the pancreas, forming a mass-like change with imaging features highly similar to pancreatic ductal adenocarcinoma (6). The diagnostic difficulty of IgG4-related pancreatitis stems primarily from the high degree to which its morphological presentation mimics malignancy. Imaging typically provides the earliest indication of pancreatic abnormalities, and the space-occupying features presented often carry high weight in the diagnostic pathway (7). The unique pathophysiological mechanism of IgG4-RD—namely, immune-mediated progressive fibrosis and inflammatory cell infiltration—is the root cause of its imaging presentation as a space-occupying lesion mimicking malignancy. Although previous studies have summarized some imaging signs that may suggest IgG4-AIP, such as delayed enhancement, long-segment stenosis of the pancreatic and bile ducts, and a capsule-like low-density rim around the pancreas, these features are not specific and are not present in all cases simultaneously. In the absence of clear evidence of extra-pancreatic involvement, the aforementioned imaging clues are often insufficient to effectively distinguish inflammatory lesions from malignancy in the preoperative stage. Theoretically, histological examination can provide key evidence for differential diagnosis; however, sampling of pancreatic lesions is limited by anatomical location and procedural risks. Small-sample biopsies often fail to fully capture the typical pathological features of IgG4-RD. Histological results in some cases may only show non-specific inflammation or fibrotic changes, which neither clearly support a diagnosis of IgG4-related disease nor rule out the possibility of malignancy at the initial stage. Consequently, the inflammatory nature of IgG4-related pancreatitis is often obscured by morphological manifestations early in the diagnostic workflow. Previous literature reports indicate that a subset of IgG4-related pancreatitis cases are ultimately diagnosed only after systematic pathological and immunohistochemical analysis following the acquisition of a complete resection specimen (8). These cases do not reflect the rarity of the disease itself, but rather suggest that when IgG4-RD appears as a focal pancreatic lesion, its clinical and imaging presentations can dominate the diagnostic pathway for an extended period, preventing the immune-mediated mechanism from being fully recognized in the early stages (9, 10).

Morphologically, IgG4-AIP can be classified into two distinct subtypes: a diffuse form, characterized by generalized pancreatic enlargement with a sausage-shaped appearance, and a focal mass-forming type, which presents as a discrete, localized lesion within the pancreas. The diffuse form is the more commonly recognized pattern and is often accompanied by classic imaging features such as a capsule-like rim and loss of pancreatic lobularity. In contrast, the focal mass-forming type accounts for approximately 40% of IgG4-AIP cases and constitutes the primary source of diagnostic confusion with pancreatic ductal adenocarcinoma. The focal mass-forming type lacks the characteristic diffuse enlargement and instead produces a localized mass, most frequently in the pancreatic head, that closely mimics the morphological and hemodynamic features of a malignant neoplasm on cross-sectional imaging. Diagnostically, the International Consensus Diagnostic Criteria (ICDC) for autoimmune pancreatitis classify IgG4-AIP as type 1 AIP, requiring a combination of parenchymal imaging features, serological evidence (elevated serum IgG4 ≥ 135 mg/dL), other organ involvement, histopathological findings (lymphoplasmacytic sclerosing pancreatitis, or LPSP), and response to steroid therapy. However, when the disease presents in its focal mass-forming variant without diffuse enlargement or obvious extra-pancreatic manifestations, meeting these composite criteria preoperatively becomes significantly more challenging, as the imaging criterion alone is often indeterminate. This diagnostic gap is the fundamental reason why a disproportionate number of focal mass-forming IgG4-AIP cases are ultimately diagnosed only after surgical resection.

This paper reports a case of IgG4-related sclerosing disease involving the pancreas, where the patient presented with a focal mass in the pancreatic head and biliary obstruction. Preoperative assessment highly suspected pancreatic malignancy, but the diagnosis was finally clarified through pathological and immunohistochemical examination of the surgically resected specimen (11, 12). By reviewing the complete diagnosis and treatment process of this case and systematically reviewing previous relevant literature, this paper aims to analyze the diagnostic difficulties of IgG4-related pancreatitis, hoping to provide a reference for comprehensive judgment in similar clinical scenarios.

## Case report

A 70-year-old male patient was admitted with a complaint of “upper abdominal bloating for more than 2 months.” The patient had experienced intermittent upper abdominal pain over the past 2 months, which progressively worsened, accompanied by abdominal bloating and darkening of urine. There were no significant symptoms of nausea, vomiting, fever, chills, or gastrointestinal bleeding. During the course of the illness, there was no noticeable weight loss or significant reduction in appetite.

Regarding the patient’s medical history, he was diagnosed with type 2 diabetes mellitus 2 days prior to hospitalization, but had not yet received systemic treatment. He had no history of hepatitis, tuberculosis, or other infectious diseases. Additionally, he had no history of hypertension, coronary heart disease, or other chronic conditions. There was no history of previous surgeries or trauma. Family medical history was unremarkable. The patient reported a 15-year history of active smoking and alcohol consumption, which he has not discontinued.

Upon physical examination, the patient’s vital signs were stable. No obvious jaundice was noted in the sclera. Abdominal examination revealed mild tenderness in the upper abdomen without rebound tenderness or muscle rigidity. No clear mass was palpated, and the liver and spleen were not palpable below the costal margin. Murphy’s sign was negative.

After admission, laboratory tests revealed significant cholestatic liver dysfunction with multiple abnormal values, which were systematically summarized in Table 1. Among the results, transaminases, bilirubin, alkaline phosphatase, gamma-glutamyltransferase, and total bile acids were markedly elevated. Tumor marker tests showed a significant increase in CA19-9, while AFP, CEA, and other markers remained within the normal range.

**Table 1:** Laboratory biochemical parameters and tumor marker results upon admission.

Item	Result	Unit
Basophils	1.1	%
CRP	6.77	mg/L
AMY	226	U/L
Pancreatic Amylase	221	U/L
Lipase	498.5	U/L
AFP	3.7	ng/ml
CEA	4.4	ng/ml
CA19-9	494.0	U/ml
HbA1c	8.96	%
ALT	431	U/L
AST	310	U/L
TBIL	52.2	umol/L
DBIL	30.3	umol/L
ALB	42.7	g/L
TBA	133.3	umol/L
AKP	339	U/L
GGT	2,373	U/L

**Figure 1:**
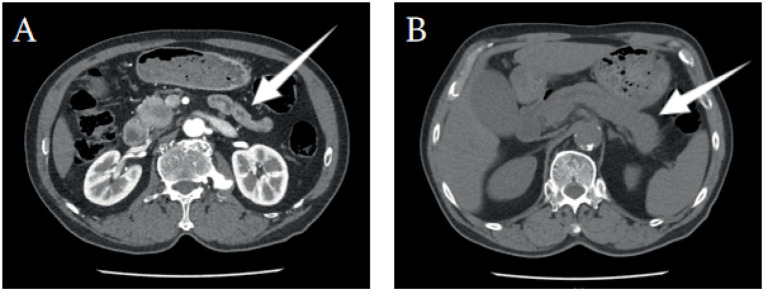
Upper Abdominal Enhanced CT Images. **(A)** Arterial phase CT scan showing enlargement of the uncinate process of the pancreas with uneven density, presenting patchy low-density areas. The common bile duct and main pancreatic duct are obstructed, and the adjacent bile duct and duodenal wall are thickened. The contrast-enhanced scan shows mild, uneven enhancement. **(B)** Venous phase CT scan showing a mass-like lesion in the uncinate process of the pancreas, with pancreatic parenchymal enlargement and dilatation of the bile ducts.

**Figure 2:**
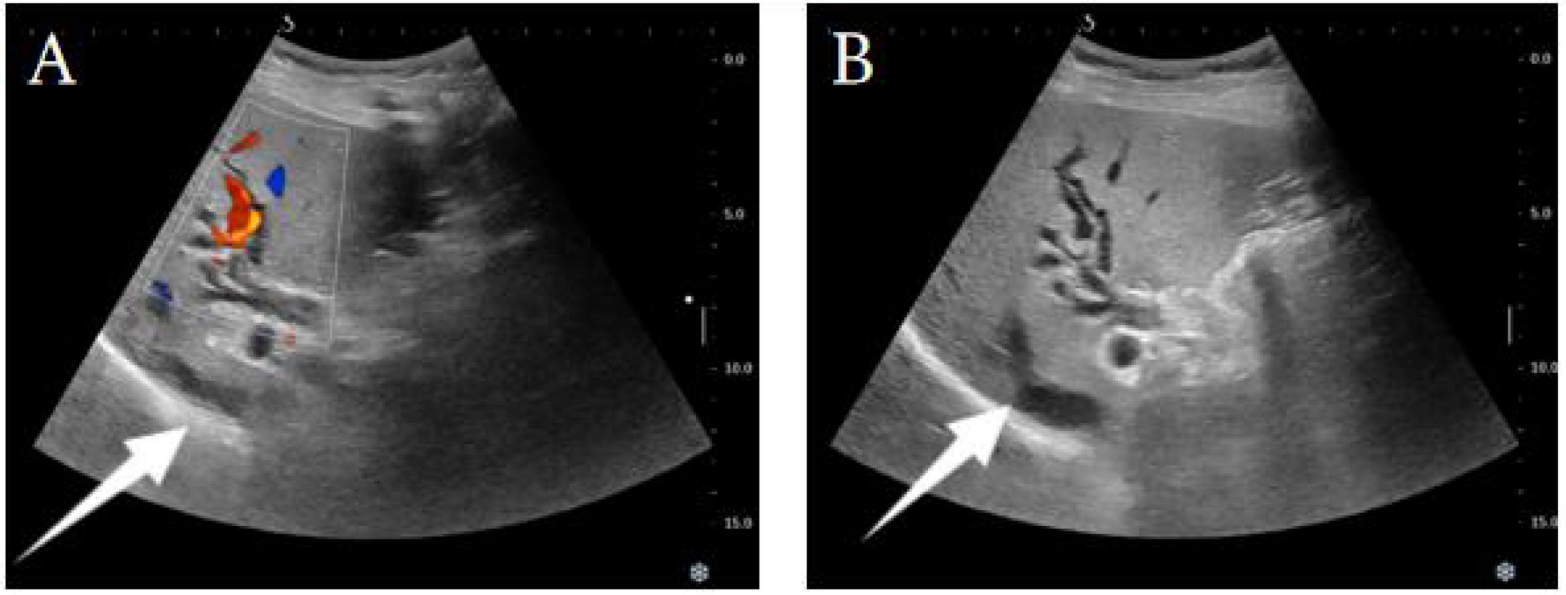
Upper abdominal ultrasound images. **(A)** Focal lesion in the pancreatic head region with dilatation of intra- and extra-hepatic bile ducts. Color Doppler imaging shows abnormal blood flow around the mass. **(B)** Focal lesion in the pancreatic head region with significant bile duct dilatation. No obvious abnormal blood flow around the mass is seen.

Abdominal CTA + CTV revealed an enlargement of the uncinate process of the pancreas with uneven density, presenting patchy low-density areas and blurred boundaries. The common bile duct and main pancreatic duct were obstructed at the lesion site, with thickening of the adjacent bile duct and duodenal wall. The contrast-enhanced scan showed mild uneven enhancement, resulting in dilatation of the common bile duct, intra- and extra-hepatic bile ducts, and the main pancreatic duct. This suggested a mass-like lesion in the uncinate process of the pancreas involving the common bile duct and adjacent duodenum, raising concern for a neoplasm. Pancreatic blood supply also showed evidence of a mass in the pancreatic head. Ultrasound examination revealed dilatation of the intra- and extra-hepatic bile ducts, suggesting a mass-like lesion in the lower segment of the common bile duct or the pancreatic head. Overall, the imaging findings strongly suggested a potential pancreatic or periampullary tumor. Relevant imaging and ultrasound data are summarized in Figures 1 and 2. Given the patient’s advanced age, obstructive biliary symptoms, markedly elevated CA19-9, and imaging features indicative of a “tumor-like” change, it was challenging to definitively exclude malignancy preoperatively. During the initial diagnostic assessment, pancreatic ductal adenocarcinoma was heavily prioritized in the differential diagnosis. This reasoning was driven by the presence of a focal mass in the pancreatic head, significant biliary obstruction, and a markedly elevated CA19-9 level. Conversely, IgG4-related autoimmune pancreatitis was considered a low probability at that time, as the patient lacked the classic imaging features of diffuse pancreatic enlargement and did not exhibit obvious synchronous extra-pancreatic systemic manifestations.

Given the patient’s advanced age, obstructive biliary symptoms, markedly elevated CA19-9, and imaging features indicative of a ‘tumor-like’ change, it was challenging to definitively exclude malignancy preoperatively. Notably, magnetic resonance cholangiopancreatography (MRCP) and endoscopic ultrasound-guided fine-needle aspiration (EUS-FNA) were not performed during the preoperative workup. Following a multidisciplinary discussion and a thorough evaluation of surgical risks, the clinical consensus leaned heavily toward an upfront surgical approach due to the high index of suspicion for a resectable malignancy. The patient and his family were informed, and a pancreaticoduodenectomy (Whipple procedure) was performed on November 6, 2024, under general anesthesia.

**Figure 3:**
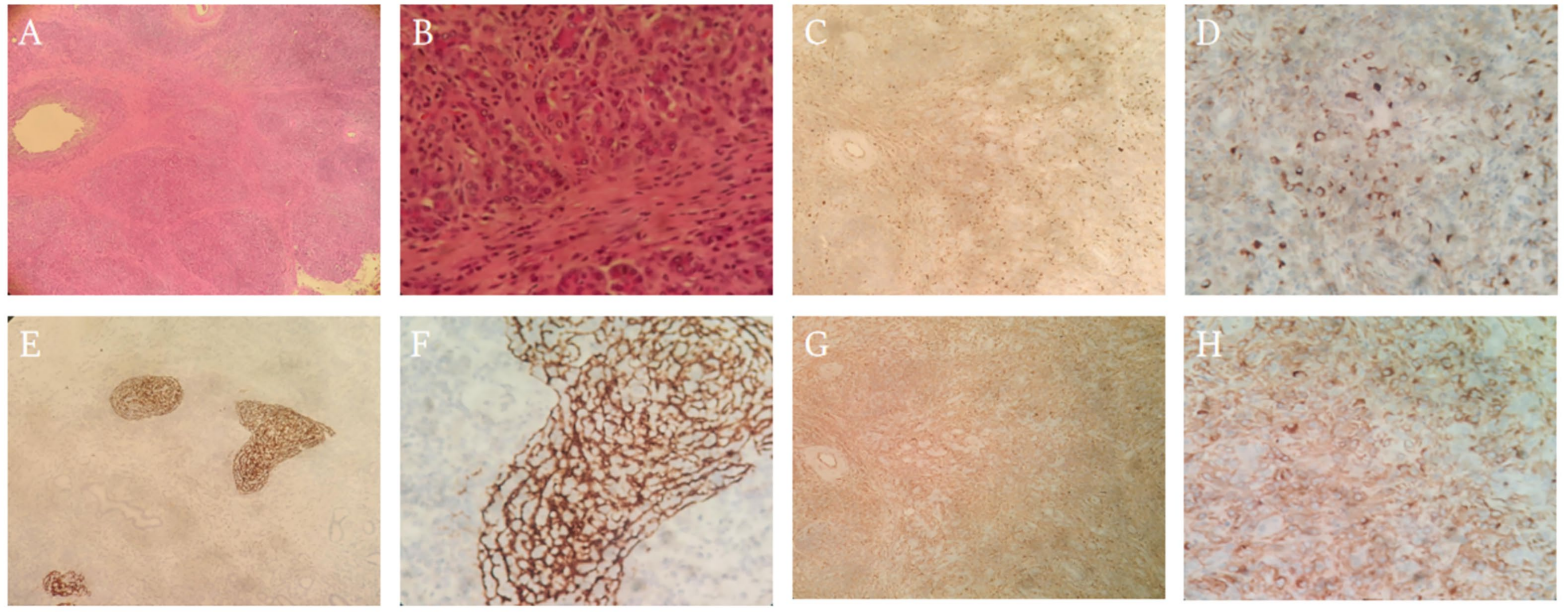
Pathological examination and immunohistochemistry. This figure shows the pathological findings of the patient’s pancreatic and bile duct tissues. **(A, B)** are 40× and 100× hematoxylin and eosin (HE) staining images, respectively, showing extensive lymphoplasmacytic infiltration in the pancreatic and bile duct tissues, with noticeable fibrosis in certain areas, indicating the presence of an inflammatory response. **(C, D)** Are 100× and 200× IgG4 staining images, revealing a significant increase in IgG4-positive plasma cells, with plasma cell aggregation clearly visible under 200× magnification. These features support the diagnosis of IgG4-related sclerosing disease. **(E, F)** are 100× and 200× CD21 staining images, showing B cell infiltration, with the distribution and activity of B cells further supporting the involvement of immune responses. Finally, **(G, H)** are 100× and 200× IgG staining images, demonstrating the deposition of immunoglobulin in the lesion area. The widespread distribution of IgG under 200× magnification aligns with the IgG4 staining results, further reinforcing the immunological evidence of the inflammatory response.

Macroscopic examination revealed thickening and rigidity of the pancreatic and bile duct walls at the junction, with prominent fibrotic changes and focal mucinous degeneration. Histological analysis showed extensive lymphoplasmacytic infiltration with significant fibrosis in both pancreatic and bile duct tissues. The bile duct walls were markedly thickened, with no clear malignant tumor components identified. Immunohistochemistry revealed a significant increase in IgG4-positive plasma cells (>50 per high-power field), with an IgG4/IgG ratio greater than 40%, positive CD38 and CD138 staining, low Ki-67 proliferation index, and negative EBER. These findings were suggestive of IgG4-related sclerosing disease. Relevant histological and immunohistochemical images are shown in Figure 3. Based on the clinical presentation, imaging findings, and pathological features, the final diagnosis was IgG4-related sclerosing disease leading to autoimmune pancreatitis, with involvement of the biliary system.

The patient recovered smoothly after surgery, with liver function and bilirubin levels gradually improving. No severe complications were observed. During the postoperative follow-up period, the patient demonstrated an excellent overall prognosis. Routine laboratory evaluations confirmed that both his CA19-9 and total bilirubin levels had completely returned to normal reference ranges.

Given the confirmed diagnosis of IgG4-related sclerosing disease, the postoperative management plan included close surveillance for potential disease relapse or involvement of other organs. Although the primary lesion was completely resected, the systemic nature of IgG4-RD necessitates long-term monitoring of serum IgG4 levels, liver function, and imaging surveillance of the pancreatic remnant, biliary system, and other commonly affected organs (salivary glands, retroperitoneum, kidneys). At the time of the most recent follow-up, no evidence of disease recurrence or new organ involvement was identified, and the initiation of immunosuppressive therapy was deferred given the complete surgical resection and absence of residual disease. However, should any signs of relapse emerge during continued follow-up, the treatment plan includes prompt initiation of oral prednisone (0.6 mg/kg/day with gradual tapering) as the first-line therapeutic intervention, with rituximab reserved as a second-line option if steroid-refractory disease develops.

The patient reported that he has experienced a smooth postoperative recovery. Since the surgical intervention and up to the most recent follow-up, he noted no special abnormalities or recurrent symptoms. He expressed satisfaction with his current clinical status and profound relief regarding the benign nature of the disease. During the follow-up interview, the patient reflected on his overall experience with the disease course. He described significant psychological distress during the preoperative period, particularly upon learning of the suspected pancreatic malignancy, which he associated with a feared poor prognosis. He reported that the uncertainty regarding the nature of the lesion was the most anxiety-provoking aspect of his illness. After receiving the postoperative pathological results confirming a benign inflammatory condition, the patient expressed considerable emotional relief and described this as a “second chance.” However, he also expressed concern about the extent of the surgery he had undergone for what turned out to be a non-malignant disease, and stated that he would have preferred a less invasive diagnostic and therapeutic approach had it been available. The patient voiced hope that sharing his experience might help other patients in similar situations avoid unnecessary major surgery. He reported that his quality of life has returned to near-baseline, with only mild digestive adjustments required following the pancreaticoduodenectomy. He continues to adhere to regular follow-up appointments and laboratory monitoring as recommended. The complete clinical course of this case, from initial symptom onset to the most recent follow-up, is summarized in Table 4.

**Table 4:** Timeline of the clinical course (CARE guideline compliant).

Time point	Date/duration	Clinical events	Diagnostic and therapeutic actions
Symptom onset	Early September 2024 (~2 months before admission)	Intermittent upper abdominal pain, progressive worsening; abdominal bloating; darkening of urine	Patient sought initial medical evaluation
New-onset diabetes	2 days before admission (early November 2024)	Diagnosed with type 2 diabetes mellitus (HbA1c 8.96%)	No systemic treatment initiated at this time
Hospital admission	November 2024	Presented with upper abdominal bloating for >2 months; no jaundice on examination; mild upper abdominal tenderness	Vital signs stable; physical examination performed
Laboratory workup	Day of admission	Cholestatic liver dysfunction identified; markedly elevated CA19-9 (494.0 U/ml); elevated amylase/lipase; AFP and CEA within normal range	Complete blood biochemistry; tumor marker panel (Table 1)
Imaging assessment	Days 1–3 of admission	CTA/CTV: mass in uncinate process with uneven density and blurred boundaries; CBD and MPD obstruction; bile duct and duodenal wall thickening. Ultrasound: bile duct dilatation and mass-like lesion	Abdominal CTA + CTV (Figure 1); Abdominal ultrasound (Figure 2); MRCP and EUS-FNA not performed
Multidisciplinary discussion	Pre-surgical	High suspicion for pancreatic or periampullary malignancy; IgG4-AIP considered low probability due to absence of diffuse enlargement and extra-pancreatic manifestations	Decision for upfront surgical resection; serum IgG4 level not tested preoperatively
Surgical intervention	November 6, 2024	Pancreaticoduodenectomy (Whipple procedure) performed under general anesthesia	Informed consent obtained; complete surgical resection
Pathological diagnosis	Post-surgery (days)	Macroscopic: thickening and rigidity of pancreatic/bile duct walls; fibrotic changes. Histology: dense lymphoplasmacytic infiltration; significant fibrosis; no malignant components	IHC: IgG4+ plasma cells >50/HPF; IgG4/IgG >40%; CD38+; CD138+; Ki-67 low; EBER negative (Figure 3)
Final diagnosis	Post-surgery	IgG4-related sclerosing disease with autoimmune pancreatitis and biliary involvement confirmed	Diagnosis established per ICDC/ACR-EULAR criteria based on histopathological and immunohistochemical evidence
Postoperative recovery	Weeks following surgery	Smooth recovery; liver function and bilirubin levels gradually normalized; CA19-9 returned to normal range; no severe complications	Routine laboratory monitoring; surveillance plan for IgG4-RD recurrence initiated
Latest follow-up	Most recent visit (2025)	Patient reports no recurrent symptoms or abnormalities; quality of life near-baseline with mild digestive adjustments; patient expresses relief and satisfaction	Continued monitoring of serum IgG4, liver function, and imaging of pancreatic remnant and biliary system; immunosuppressive therapy deferred

## Discussion

IgG4-related sclerosing disease is a systemic immune-mediated disease characterized by inflammation and fibrosis (3). When involving the pancreas, it primarily manifests as IgG4-related pancreatitis. In clinical practice, the disease does not always present with typical inflammatory features in the early stages of pancreatic involvement (7). Some patients may present with focal, mass-like changes that highly resemble pancreatic tumors on imaging, leading to their prioritization within the tumor differential diagnosis during initial assessment. The patient in this case exhibited these characteristics; he was highly suspected of having a pancreatic tumor during the initial visit due to focal space-occupying changes in the pancreatic head, and the diagnostic pathway subsequently revolved around excluding malignancy. It was only with the clarification of subsequent pathology results and follow-up information that it was retrospectively recognized that the patient’s pancreatic lesion was actually an inflammatory change caused by IgG4-related sclerosing disease (8). Therefore, IgG4-related pancreatitis may be incorporated into the diagnostic pathway for pancreatic tumors in the early stages due to mass-like manifestations, complicating the subsequent diagnosis and management process.

The present case is particularly noteworthy because it represents a focal mass-forming variant of IgG4-AIP, which is the subtype most frequently misdiagnosed as pancreatic malignancy. While the diffuse form of IgG4-AIP, with its characteristic sausage-shaped pancreatic enlargement, is relatively easier to recognize, the focal mass-forming variant presents a fundamentally different diagnostic challenge. This subtype accounts for approximately 40% of type 1 AIP cases and is disproportionately represented among cases that proceed to unnecessary surgical resection. The clinical novelty of the current case lies in the comprehensive documentation of how every major diagnostic parameter including imaging morphology, tumor marker elevation (CA19-9), clinical presentation, and the absence of systemic features converged to simulate pancreatic cancer in a focal mass-forming IgG4-AIP presentation. This convergence of misleading features, combined with the availability of a complete surgical specimen for definitive histopathological characterization, provides a uniquely instructive example for understanding the diagnostic pitfalls specific to this subtype.

According to the 2019 American College of Rheumatology/European League Against Rheumatism (ACR/EULAR) classification criteria for IgG4-RD, a definitive diagnosis requires a comprehensive integration of clinical, serological, radiological, and pathological data. In the context of our patient, the clinical management was primarily driven by the focal uncinate process mass and a markedly elevated CA19-9 level (494.0 U/mL), which strongly skewed the preoperative judgment toward malignancy and overshadowed potential inflammatory clues. Crucially, a preoperative assessment for synchronous extra-pancreatic organ involvement—such as testing serum IgG4 levels or evaluating the salivary glands and retroperitoneum—was not performed. Retrospectively, securing such systemic data could have provided critical diagnostic weight to pivot the clinical suspicion toward IgG4-RD. Ultimately, the postoperative histological findings of dense lymphoplasmacytic infiltration, extensive fibrosis, and >50 IgG4-positive plasma cells per high-power field with an IgG4/IgG ratio >40% perfectly aligned with the ACR/EULAR histopathology criteria, confirming the diagnosis. This highlights a significant clinical pitfall: when managing highly suspicious localized pancreatic masses, the omission of systematic screening for other organ involvement can lead directly to overtreatment.

To further clarify the diagnostic reasoning in this case, it is instructive to systematically compare the diagnostic criteria fulfilled for both pancreatic ductal adenocarcinoma (PDAC) and focal mass-forming IgG4-AIP. For PDAC, the clinical features supporting this diagnosis included: (1) the patients age of 70 years, within the peak incidence demographic; (2) a focal mass in the pancreatic head with ill-defined borders on CT; (3) biliary obstruction with markedly elevated CA19-9 (494.0 U/mL); (4) new-onset diabetes mellitus; and (5) mild uneven enhancement pattern on contrast CT. Conversely, features retrospectively supporting IgG4-AIP included: (1) the absence of vascular invasion or encasement, which is atypical for PDAC at this size; (2) thickening of the bile duct and duodenal wall suggesting an inflammatory rather than infiltrative process; (3) the delayed enhancement pattern, which is more characteristic of fibro-inflammatory tissue than of desmoplastic tumor stroma; and (4) the lack of upstream pancreatic atrophy, which is commonly seen in PDAC with ductal obstruction. By the ICDC criteria for definitive type 1 AIP diagnosis, the following were ultimately confirmed postoperatively: Level 1 histological evidence (LPSP with >10 IgG4-positive cells/HPF, here >50/HPF), and Level 1 serological evidence (IgG4/IgG ratio >40%). However, these critical data points were not available preoperatively, and the imaging features alone were classified as indeterminate (Level 2 at best) due to the focal rather than diffuse nature of the lesion. This analysis demonstrates that the focal mass-forming variant of IgG4-AIP can satisfy multiple clinical criteria for PDAC simultaneously, and that the definitive diagnostic features of IgG4-AIP (histological and serological) often require tissue acquisition to be confirmed, underscoring the central importance of EUS-guided biopsy in this clinical scenario.

In the early stages of pancreatic involvement, IgG4-related sclerosing disease often lacks diagnostic evidence that can directly support its inflammatory nature, creating significant uncertainty for initial clinical judgment (13). Imaging assessment plays a crucial role in the initial evaluation of pancreatic lesions, but it primarily reflects morphological changes and has objective limitations in distinguishing inflammatory fibrosis from neoplastic lesions. IgG4-related pancreatitis can manifest as diffuse or focal pancreatic enlargement; when involvement is focal, it is particularly easy to confuse with pancreatic ductal adenocarcinoma (5, 9). Some cases show delayed enhancement on contrast scans, and when the pancreatic and bile ducts are involved, they often present morphological features of long-segment stenosis rather than abrupt interruption. Reports also describe a “capsule-like” low-density rim around the pancreas. However, these imaging findings are not specific signs, and not all patients possess them simultaneously; thus, a single imaging feature is difficult to reliably distinguish inflammatory lesions from malignancy in the early stages. In clinical practice, when a focal lesion highly mimics malignancy, imaging often assumes the role of risk assessment rather than qualitative diagnosis. Meanwhile, evidence directly supporting an inflammatory diagnosis is often relatively insufficient in the early stages: serological abnormalities lack sufficient specificity, and other clues of systemic involvement do not necessarily appear synchronously. Even when histological sampling is attempted, limited by the safety of pancreatic sampling and specimen representativeness, small-sample pathology results may only show non-specific inflammation or fibrotic changes, failing to form a clear direction in the initial assessment phase. Under this evidence structure, the inflammatory characteristics of IgG4-related pancreatitis are often difficult to prioritize, and the diagnostic process is consequently more likely to proceed along tumor-related judgment pathways (1, 5, 7, 10, 14).

Histological evidence is theoretically regarded as an important basis for distinguishing inflammatory lesions from tumors (7). From a histological perspective, IgG4-related sclerosing disease has relatively characteristic pathological features, including dense lymphoplasmacytic infiltration, storiform fibrosis, and obliterative phlebitis, often accompanied by an increased number of IgG4^+^ plasma cells. However, during the actual sampling of focal pancreatic lesions, limited by anatomical location, safety, and sample volume, small-sample biopsies often fail to fully present the above typical features. Pathological results in some cases may only show non-specific inflammation or fibrotic changes, which are neither sufficient to clearly support a diagnosis of IgG4-related disease nor effective in ruling out the possibility of malignancy at the initial stage (15). Therefore, histological evidence in the early assessment of IgG4-related pancreatitis needs to be interpreted comprehensively in conjunction with the clinical context and follow-up information, rather than serving as a single decisive basis. Its practical application in IgG4-related pancreatitis is restricted by multiple conditions (9, 16). Pancreatic lesion sampling is often constrained by anatomy, procedural risk, and available tissue volume. Small-sample biopsies in some cases are difficult to fully present the histological features required for IgG4-related disease (8, 10, 17). The obtained results may only manifest as non-specific inflammation or fibrosis, which is insufficient to clearly support an inflammatory diagnosis or effectively overturn the existing tumor hypothesis at the initial stage. In this situation, histological examination does not necessarily significantly reduce diagnostic uncertainty, and its results require comprehensive interpretation combined with clinical background and subsequent information. This partly explains why, in some cases of IgG4-related pancreatitis, despite attempts to obtain histological evidence, the diagnostic pathway continues to advance along tumor-related logic (2, 7, 15, 18).

It is important to emphasize that tissue biopsy, particularly via endoscopic ultrasound (EUS), remains the gold standard for diagnostic accuracy when differentiating pancreatic adenocarcinoma from inflammatory pancreatic diseases. In the present case, EUS-FNA was not performed preoperatively. Furthermore, preoperative biliary drainage via a pancreatic or biliary stent was not considered because the patient’s total bilirubin level (52.2 umol/L), although indicative of cholestasis, did not reach the critical threshold necessitating emergency decompression. The multidisciplinary team prioritized immediate surgical resection to avoid potential infectious complications and delays associated with preoperative stenting. Retrospectively, however, performing an endoscopic retrograde cholangiopancreatography (ERCP) with stent placement to collect cytological or tissue samples from the bile duct could have been a highly valuable diagnostic step to potentially avoid unnecessary extensive surgery.

When existing examination results are still difficult to support an inflammatory diagnosis, even if there are other clues suggesting the possibility of a systemic disease, they are often difficult to gain sufficient diagnostic weight in the initial assessment phase (6, 9, 11, 19). The multi-organ involvement of IgG4-related sclerosing disease has heterogeneity in time and presentation; relevant abnormalities do not necessarily appear synchronously with pancreatic involvement, nor do they always form a clear, integrable clinical picture in the early stages. In the assessment process driven mainly by focal pancreatic lesions, such scattered and non-specific clues are more easily viewed as background information rather than key evidence for redefining the main diagnostic direction. Relevant cases in previous literature also show that systemic features are often only re-incorporated into the overall understanding of the disease nature after pathology results are clarified or gradually revealed during follow-up. This phenomenon provides a realistic background for understanding why IgG4-related pancreatitis is repeatedly interpreted as a tumor in the early stages, and also provides a logical basis for the compilation of the case data listed earlier (20).

Combining the diagnosis and treatment process of this case, we reviewed and organized relevant cases reported in the past (21–29) (Table 2). The common clinical feature of these cases is that IgG4-related pancreatitis mostly entered the diagnostic process with mass-like manifestations in the initial stage, thereby being included in pancreatic tumor-related assessments (14, 16). Although specific presentations and management strategies varied, their immune-related nature was often only gradually clarified in subsequent pathology results or follow-up processes.

**Table 2:** Review and characteristic analysis of IgG4-related pancreatitis cases mimicking pancreatic malignancy

No.	Literature (author-year)	Age/sex	Main presentation	Localization/imaging key points	CA19-9	Serum IgG4	Sampling/chain of evidence (basis for final diagnosis)	Treatment and outcome
1	Hammami et al. (2011) (37)	27/M	Progressive jaundice, mild epigastric discomfort	Imaging: "pancreatic mass/suspected carcinoma" → Intraoperative palpable head mass	433 U/ml	NR	Whipple resection specimen: Chronic pancreatitis with lymphoplasmacytic infiltration → AIP	Post-Whipple prednisone (40mg/d) with tapering; labs recovered in 3 weeks; stopped after ~10 months
2	Matsumoto, 2011 (22)	79/M	Epigastric pain; previously considered "pancreatitis due to pancreatic tail tumor"”	Pancreatic tail mass (40 × 23 mm); ERCP showed main pancreatic duct obstruction; EUS hypoechoic	Normal	256 mg/dl	EUS-FNA: No carcinoma seen; Final distal pancreatectomy specimen: IgG4+ plasma cells	Distal pancreatectomy; stable postoperative course
3	Thompson et al. (2017) (38)	57/M	Jaundice; multi-organ clues (eyes/glands/skin/prostate, etc.)	Pancreatic head mass + Double duct sign; Combined with renal wedge-shaped lesions, aortic wall thickening, generalized lymphadenopathy	Normal	1,980 mg/dl	EUS-FNAC: Cancer not confirmed; Biopsy of "inguinal lymph node" showed IgG4-RD features (IgG4+ plasma cells)	Intravenous steroid therapy; significant improvement (avoided pancreatic surgery)
4	Franchello, 2014 (24)	46/M	Jaundice, fatigue, weight loss	Pancreatic head mass (42×28mm), suspected "borderline resectable carcinoma"; accompanied by bilateral renal "pseudo-nodular changes"	Negative	180 mg/dl	EUS-FNA: No carcinoma seen but inconclusive; Renal biopsy: IgG4-related interstitial nephritis → Supported systemic IgG4-RD	Pancreatic/renal lesions shrank 1 month post-steroids; pancreatic lesion disappeared at 6 months
5	Cao, 2015 (25)	45/M	RUQ pain + Jaundice	Focal lesion in pancreatic head; Homogeneous enhancement in delayed phase on MRI; Long-segment stenosis of main pancreatic/bile ducts	53.57 U/ml (Mildly elevated)	1300 mg/dl	Proceeded directly to Whipple for "malignant pancreatic obstruction"; Resection specimen IgG4 staining: IgG4+ plasma cells up to 110/HPF	Key value in "retrospective imaging clues"; subsequent steroid regimen details NR
6	Paramythiotis, 2024 (26)	58/M	Painless obstructive jaundice (2 days)	CT showed diffuse enlargement + inflammatory changes of pancreas (esp. head); Biliary dilation	1.87 U/ml	NR	Focal AIP morphologically mimicking pancreatic head cancer	Benefited after steroid therapy
7	Parente, 2021 (27)	68/M	Significant weight loss over 10 months; poorly controlled diabetes; later developed obstructive jaundice	"Spheroid-like" mass (4.3cm) in pancreatic head; Abrupt cutoff at distal bile duct	50.8 U/ml	NR	Article emphasizes "received more radical surgical management due to pseudo-tumoral presentation"; metabolic/symptom improvement after subsequent steroids	Benefited after steroid therapy (esp. metabolism and disease control); follow-up details NR
8	Mohamed, 2024 Case 1 (28)	66/M	Jaundice, weight loss, pruritus	Pancreatic head mass + Biliary obstruction; bilateral perinephric lesions and other systemic clues observed	NR	4.6 g/L	EUS-guided biopsy showed no definite cancer; MDT managed as IgG4-AIP	Prednisolone 40mg/d effective; Azathioprine added after relapse upon tapering; Imaging showed lesion remission/regression
9	Mohamed, 2024 Case 2 (28)	73/F	Asymptomatic, only liver enzyme abnormalities	CT showed pancreatic head mass causing CBD dilation; EUS/ERCP suggested possible malignancy but cytology non-diagnostic	NR	3.84 g/L	Cytology non-diagnostic; Combined IgG4 elevation and tissue "fibrosis + chronic inflammation" favored IgG4-related lesion	"Whipple vs Steroids" option provided, patient chose steroids; mass regression on follow-up supported IgG4-AIP
10	Khan, 2024 Case 1 (29)	72/M	Jaundice (scleral icterus), clay-colored stool, etc.	Heterogeneous space-occupying lesion in pancreatic head/uncinate process; Low FDG on PET-CT; previously reported as "tumor + lung metastasis?"	42.07 U/mL	2,220 mg/L	Whipple resection specimen: No malignancy; Combined with significant IgG4 elevation → IgG4-AIP	Postoperative steroids added; CT normal at 1 year; IgG4 decreased but remained high (1470 mg/L)
11	Khan, 2024 Case 2 (29)	52/M	Referral (discovered during "fatty liver/renal cyst" checkup)	Mildly enhancing soft tissue mass in pancreatic head/uncinate process, completely encasing SMA; No biliary dilation	NR	1,130 mg/L	Maging + IgG4 elevation supported IgG4-related pancreatic lesion (Pancreatic sampling not mentioned)	Steroid therapy; No follow-up imaging (asymptomatic, did not re-examine)
12	Khan, 2024 Case 3 (29)	35/M	Epigastric pain for 2 days	Mass in pancreatic head/uncinate process encasing SMA; Progression and involvement of portal vein/hepatic artery on follow-up 4 years later	162 U/mL	2960 mg/L	CT-guided transhepatic core biopsy: Suggested periductal inflammation + fibrosis, IgG4 + plasma cells seen	Article emphasizes "steroids should be the mainstay"
13	Present case	70/M	Upper abdominal bloating for more than 2 months; new-onset type 2 diabetes diagnosed 2 days before admission; no jaundice on examination; mild upper-abdominal tenderness	Enlarged uncinate process of pancreatic head, heterogeneous density with patchy low-density shadows, blurred boundaries; Obstruction of common bile duct and main pancreatic duct at the lesion site	494U/mL	Not tested preoperatively	Whipple resection specimen: dense lymphoplasmacytic infiltration with > 50 IgG4 + plasma cells/HPF and IgG4/IgG ratio > 40% → IgG4-related sclerosing disease	No recurrence at 1-year postoperative follow-up; corticosteroid therapy held given complete surgical resection

In addition, we supplemented a series of representative reports describing IgG4-related sclerosing disease involving other organs with tumor-like appearances (Table 3), in order to illustrate that the above diagnostic process is not specific to the pancreas (9, 24, 30–34). This compilation was not intended to compare diagnostic or therapeutic strategies across different organs, but rather to serve as contextual background, highlighting that the phenomenon whereby inflammatory diseases are interpreted as neoplastic lesions when presenting with focal involvement appears to have a certain degree of generality across the spectrum of IgG4-related disease.

**Table 3:** Summary of representative literature on IgG4-related sclerosing disease involving extra-pancreatic organs presenting with tumor-like changes

Author, year	Organ involved	Initial suspected malignancy	Key misleading features	Diagnostic clue beyond primary organ	Definitive diagnostic method	Clinical implication
Sulieman, 2018 (23)	Retroperitoneum, kidney, LN, prostate	Metastatic pancreatic cancer/lymphoma	Multiple organ masses + double duct sign + lymphadenopathy	Lesion distribution does not follow a single metastatic route	Inguinal LN biopsy + IgG4 IHC	Systemic distribution is more important than "mass size"
Franchello, 2014 (24)	Kidney	Renal metastasis/RCC	Pancreatic head mass + renal parenchymal abnormality	Renal lesions are inconsistent with metastasis	Renal biopsy → IgG4-RD	Peripheral organs may give answers before the pancreas
Zen et al. (2004) (39)	Bile duct	Cholangiocarcinoma	Bile duct stenosis, progressive jaundice	Coexisting pancreatic/other organ IgG4 clues	Bile duct histology + IgG4 IHC	IgG4-SC can completely mimic cholangiocarcinoma
Hirano et al. (2006) (40)	Retroperitoneum	Retroperitoneal sarcoma	Retroperitoneal mass, progressive enlargement	Coexisting pancreas/gland involvement	Surgical biopsy + IgG4 IHC	Retroperitoneal "tumor" does not equate to sarcoma
Khosroshahi et al. (2011) (41)	Lung	Lung cancer	Pulmonary mass, FDG uptake	Coexisting gland/pancreas abnormality	Lung biopsy → IgG4-RD	IgG4-RD can present as an inflammatory pseudotumor
Kamisawa et al. (2010) (42)	Salivary gland	Salivary gland tumor	Glandular enlargement, nodular changes	Coexisting pancreatic lesions	Gland biopsy + IgG4 IHC	Head and neck masses may belong to the same disease spectrum
Deng et al. (2015) (43)	Aorta (periaortitis)	Malignant aortic tumor	Periaortic soft tissue encasement	Simultaneous pancreas/kidney involvement	Periaortic biopsy → IgG4-RD	"Vascular encasement tumor-like changes" need to be cautious

In cases where imaging, histology, and systemic clues are difficult to form a clear judgment in the early stages, some cases will further discuss whether there are other ways to assist in identifying inflammatory lesions. It is against this background of diagnostic uncertainty that glucocorticoids are mentioned in the clinical context of IgG4-related pancreatitis (12, 13). It should be pointed out that hormone-related discussions are not based on their definitive value as a differential diagnostic tool, but rather stem from clinical exploration of potential inflammatory mechanisms in the absence of reliable counter-evidence. However, in the context of pancreatic involvement presenting with tumor-like changes, the response to hormones itself lacks exclusivity, and its clinical or imaging changes in the short term are difficult to serve as a basis for clearly distinguishing inflammation from tumors. Furthermore, before the risk of malignancy is fully assessed, the potential risk of diagnostic delay caused by hormone intervention imposes strict boundaries on its application. Therefore, the discussion of glucocorticoids in such cases should be understood as a background option under diagnostic dilemmas, rather than a routine judgment path in the initial assessment phase (15, 18).

Regarding the standard treatment protocol for IgG4-related autoimmune pancreatitis, glucocorticoids remain the first-line therapy. The recommended initial regimen consists of oral prednisone at a dose of 0.6–1.0 mg/kg/day (typically 40 mg/day) for 2–4 weeks, followed by gradual tapering by 5 mg every 1–2 weeks over 3–6 months. In many patients, particularly those with relapsing disease, low-dose maintenance therapy (2.5–5 mg/day) may be continued for up to 3 years to reduce the risk of relapse. Response to steroid therapy is typically rapid and dramatic, with significant improvement in symptoms, imaging findings, and serological markers (including normalization of serum IgG4 levels) within 2–4 weeks, which also serves as a supportive diagnostic criterion. For patients who are steroid-refractory, steroid-dependent with frequent relapses, or unable to tolerate glucocorticoids, steroid-sparing immunosuppressive agents such as azathioprine (2–2.5 mg/kg/day), mycophenolate mofetil, or methotrexate may be employed. More recently, B-cell depletion therapy with rituximab (375 mg/m2 weekly for 4 doses or 1,000 mg on days 1 and 15) has demonstrated excellent efficacy in refractory IgG4-RD and is increasingly recognized as a viable second-line option. In the present case, the patient underwent surgical resection before the diagnosis of IgG4-AIP was established; therefore, glucocorticoid therapy was not initiated preoperatively. Had the correct diagnosis been established prior to surgery, a trial of corticosteroid therapy would have been the appropriate first-line approach, potentially sparing the patient from a major pancreaticoduodenectomy.

In clinical situations where IgG4-related sclerosing disease involves the pancreas and manifests as pancreatitis, diagnostic difficulties stem more from the inconsistency in the timing of acquisition and diagnostic weight of different types of evidence. Imaging abnormalities usually appear earliest and have a dominant influence on the diagnostic direction in the initial assessment phase; histological evidence is limited by sampling conditions and specimen representativeness and may not clearly reflect the inflammatory nature at the same stage; relevant clues of systemic involvement often emerge gradually during the course of the disease or follow-up; and glucocorticoid-related responses mostly have explanatory significance in post-hoc analysis. The systemic inflammatory features of IgG4-related sclerosing disease are difficult to fully recognize in the early stages of pancreatic involvement, and its pancreatitis manifestations are therefore often only clarified after subsequent evidence is gradually integrated (1, 5, 12–14).

Based on the diagnosis and treatment process of this case and previous knowledge of IgG4-related sclerosing disease, in future scenarios involving similar pancreatic involvement presenting as focal lesions, diagnostic assessment can be more targeted around the existing multi-dimensional framework. In the initial stage, when imaging manifestations highly mimic pancreatic malignancy, routine tumor assessment procedures generally still need to be prioritized. However, if histological sampling fails to confirm malignancy, and serum IgG4 levels are abnormal or imaging presents features such as delayed enhancement or long-segment stenosis of pancreatic/bile ducts, the possibility of IgG4-related sclerosing disease involving the pancreas should be considered. On this basis, further assessment for involvement of other organs, utilizing 18F-FDG PET/CT if necessary to detect potential systemic inflammatory manifestations, helps to perfect the overall understanding of the disease entity. At the treatment level, while it has been widely confirmed that IgG4-related pancreatitis responds well to glucocorticoid therapy, the prerequisite for its application is always the full assessment and exclusion of pancreatic malignancy risks (35, 36). The experience of this case suggests that when multiple pieces of evidence gradually point to an inflammatory mechanism, avoiding the long-term fixation of focal pancreatic lesions as tumor hypotheses may help reduce unnecessary invasive treatments. The above thinking is based on limited cases and retrospective analysis; it summarizes not a fixed diagnostic path, but a practical reflection on how existing diagnostic tools and treatment strategies can be reasonably integrated in specific clinical contexts.

A notable limitation of this case report is the absence of preoperative histological sampling, such as EUS-guided biopsy, which led to a major surgical resection for a benign condition. However, a significant strength of this report is the availability of the fully resected specimen, which allowed for a comprehensive pathological and immunohistochemical analysis, providing definitive insights into the dense lymphoplasmacytic infiltration of mass-forming IgG4-RD.

## Conclusion

IgG4-related sclerosing disease involving the pancreas is a rare condition that often initially presents as a focal pancreatic mass and biliary obstruction. Its imaging and laboratory findings closely resemble those of pancreatic malignancies. In this case, the patient had clear mass-like changes in the head of the pancreas, with significant dilatation of the intra- and extra-hepatic bile ducts and an elevated CA19-9 level. Given the lack of direct evidence supporting an inflammatory lesion preoperatively, the clinical approach followed the pancreatic tumor path. This case underscores that while incorporating IgG4-RD into the routine differential diagnosis of pancreatic tumors serves as a beneficial relative clinical clue, it is not definitively crucial on its own. Diagnostic accuracy heavily relies on obtaining histological evidence, ideally through EUS-guided tissue biopsy, prior to defining the surgical strategy. When multiple lines of clinical and relative serological evidence point toward an inflammatory mechanism, rigorous preoperative tissue sampling must be prioritized. Confirming the inflammatory nature of the lesion, strictly after malignancy is excluded via biopsy, can facilitate the transition to corticosteroid therapy, thereby avoiding long-term assumptions of malignancy and preventing unnecessary invasive surgical treatments.

## Data Availability

The original contributions presented in the study are included in the article/supplementary material, further inquiries can be directed to the corresponding authors.
